# Prevalence of Vitamin D Deficiency in Children and Adolescents With Type 1 Diabetes Mellitus: A Systematic Review and Meta-Analysis

**DOI:** 10.7759/cureus.83843

**Published:** 2025-05-10

**Authors:** Kaushikkumar S Barot, Zainab A Abbasi, Gautham Varun Krishna Mohan, Syed Ashraf Abid, Syed Ahmar Hussain, Calvin R Wei, Neelum Ali

**Affiliations:** 1 Pediatrics, Shantabaa Medical College & General Hospital Amreli, Amreli, IND; 2 Pediatric Medicine, Quaid-e-Azam Medical College, Islamabad, PAK; 3 Internal Medicine, Tirunelveli Medical college, Tirunelveli, IND; 4 Medicine, Deccan College of Medical Sciences, Hyderabad, IND; 5 Medicine, Sindh Medical College, Karachi, PAK; 6 Research and Development, Shing Huei Group, Taipei, TWN; 7 Internal Medicine, University of Health Sciences, Lahore, PAK

**Keywords:** adolescents, children, meta-analysis, type 1 diabetes mellitus, vitamin d deficiency

## Abstract

Our systematic review and meta-analysis examined the proportion of vitamin D deficiency in children and adolescents with type 1 diabetes mellitus (T1DM). We conducted a comprehensive literature search across multiple databases, including PubMed, Cochrane Library, Web of Science, Ovid Medline, and Embase, for studies published between January 2016 and March 2025. Following Preferred Reporting Items for Systematic Reviews and Meta-Analyses (PRISMA) guidelines, 29 studies comprising 2516 participants were included in the final analysis. Quality assessment was performed using the Newcastle-Ottawa Scale. The pooled analysis revealed that 46% (95% CI: 34-58%) of children and adolescents with T1DM had vitamin D deficiency, with significant heterogeneity observed across studies (I² = 97.98%, p<0.01). Subgroup analysis showed geographical variations with the highest deficiency rates in Africa (74%). The definition of vitamin D deficiency also impacted results, with cutoffs of <25 ng/mL yielding the highest proportion (80%) and <12 ng/mL the lowest (14%). Despite methodological limitations, including clinical setting bias, varied study designs, and inconsistent deficiency thresholds, our findings highlight the substantial burden of vitamin D deficiency in pediatric T1DM patients. This suggests the need for routine screening and potential supplementation strategies, though further research is required to establish optimal vitamin D levels for T1DM management and determine whether supplementation could play a preventive or therapeutic role in this population.

## Introduction and background

Vitamin D plays a crucial role in bone metabolism, immune regulation, and overall health. Numerous chronic illnesses, including autoimmune conditions like type 1 diabetes mellitus (T1DM), have been linked to vitamin D deficiency [1]. Insulin insufficiency and hyperglycemia are the results of T1DM, a chronic autoimmune disease marked by the death of pancreatic beta cells [2]. There may be a connection between vitamin D levels and the etiology, development, and complications of type 1 diabetes, according to recent data [3]. 

Several studies have reported a higher prevalence of vitamin D deficiency in children and adolescents with T1DM compared to their healthy counterparts [4]. Potential mechanisms linking vitamin D deficiency to T1DM include its immunomodulatory effects, influence on pancreatic beta-cell function, and regulation of inflammatory pathways. Vitamin D is known to enhance the activity of regulatory T cells, suppress pro-inflammatory cytokines, and promote immune tolerance, which are critical factors in autoimmune diseases like T1DM [5-6]. Furthermore, vitamin D receptors (VDR) and the enzyme 1α-hydroxylase, which converts vitamin D into its active form, are expressed in pancreatic beta cells, suggesting a direct role in insulin secretion and glucose metabolism [7]. 

Despite the biological plausibility of the association between vitamin D deficiency and T1DM, reported prevalence rates vary widely across studies due to differences in study populations, geographic locations, seasonal variations, definitions of vitamin D deficiency, and assay methodologies [8-12]. The most commonly used biomarker for assessing vitamin D status is serum 25-hydroxyvitamin D (25(OH)D), with deficiency generally defined as levels below 20 ng/mL (50 nmol/L) [13]. However, some studies use different cut-off values, leading to inconsistencies in prevalence estimates. In addition, factors such as ethnicity, dietary intake, sun exposure, obesity, and genetic predisposition may contribute to variations in vitamin D levels among children and adolescents with T1DM. 

Given these discrepancies, a comprehensive synthesis of the existing literature is needed to quantify the proportion of vitamin D deficiency in children and adolescents with T1DM. Understanding the burden of vitamin D deficiency in this population is critical, as it may inform clinical guidelines for routine screening and supplementation strategies. Furthermore, identifying potential risk factors for vitamin D deficiency in T1DM could help in designing targeted interventions to mitigate complications associated with both conditions. This systematic review and meta-analysis aim to estimate the pooled proportion of vitamin D deficiency in children and adolescents with T1DM. The findings will provide valuable insights into the magnitude of vitamin D deficiency in pediatric T1DM patients and its potential clinical implications. 

## Review

Methodology 

Literature Search and Search Strategy 

A comprehensive literature search was conducted to identify published studies reporting the proportion of vitamin D deficiency in children and adolescents with type 1 diabetes (T1DM). Studies published from the 1st of January 2016 to 25 March 2025 were considered. The following electronic databases were systematically searched: PubMed, Cochrane Library, Web of Science, Ovid Medline, and Embase. The search strategy was developed using a combination of Medical Subject Headings (MeSH) terms and free-text keywords related to T1DM, vitamin D, and the pediatric population. The search strategy was refined to ensure comprehensiveness by combining MeSH terms with free-text phrases using Boolean operators (AND, OR). Additionally, the reference lists of relevant articles and systematic reviews were manually screened to identify additional eligible studies. There were no restrictions on the language of the included studies. After completing the search, all retrieved records were imported into EndNote Version X9 for reference management (Clarivate, Philadelphia, USA), and duplicates were removed before proceeding with the screening process. A manual search of reference lists from included studies and relevant systematic reviews was performed to identify additional studies. Search was performed by two authors independently. Any disagreement between the two authors in the search process was resolved through discussion. 

Study Selection 

The Preferred Reporting Items for Systematic Reviews and Meta-Analyses (PRISMA) criteria were followed in the study's execution and reporting. Two separate reviewers examined the titles and abstracts of the retrieved publications to find possibly relevant studies after eliminating duplicates. The chosen studies' full-text articles were then evaluated for inclusion using predetermined eligibility standards. A third reviewer was consulted or discussed with in order to settle any disputes between the reviewers. 

Studies were included if they met the following criteria: (1) observational studies (cross-sectional, case-control, or cohort) reporting the proportion of vitamin D deficiency in children and/or adolescents with T1DM. Studies were excluded if they: (1) focused only on adults or mixed populations without separate data for children/adolescents; (2) lacked primary data (e.g., reviews, editorials, commentaries, conference abstracts without full-text); (3) investigated only vitamin D supplementation effects rather than deficiency prevalence; or (4) did not specify the diagnostic criteria for T1DM or vitamin D deficiency. 

Data Extraction and Quality Assessment 

Data extraction from included studies was performed using Microsoft Excel (Microsoft Corporation, Redmond, USA). Data extracted from included studies were: author name, year of publication, country, study design, sample size, number of patients with vitamin D deficiency, and characteristics of participants. Data were extracted by two authors independently. Any disagreement between the two authors was resolved through discussion. Quality assessment was performed for cohort and case-control using the New-Castle Ottawa Scale (NOS). The NOS score ranged from 0 to 9. Studies scoring above 6 were classified as having reasonably high quality, those with scores between 5 and 6 were considered moderate quality, while studies scoring below 5 were categorized as low quality. The quality of cross-sectional studies was assessed using the methodology checklist developed by the Agency for Healthcare Research and Quality (AHRQ).

Statistical Analysis 

Data analysis was performed using STATA Version 17.0 (StataCorp, College Station, USA). To estimate the pooled effect, a 95% confidence interval (CI) was applied, with statistical significance set at P < 0.05. A random-effects model was used to aggregate studies reporting the proportion of vitamin D deficiency in children and adolescents with T1DM. Heterogeneity between studies was assessed using the I² statistic. If I² was below 50%, heterogeneity was considered low, and a fixed-effects model was employed. However, if I² exceeded 50%, indicating substantial heterogeneity, a random-effects model was implemented. We performed subgroup analysis based on the year of publication, i.e., before 2020 and 2020 onwards, study design, and continent (Asia, Europe, Africa, America). Publication bias was assessed using the Funnel plot and the Egger's test.

Results 

**Figure 1:**
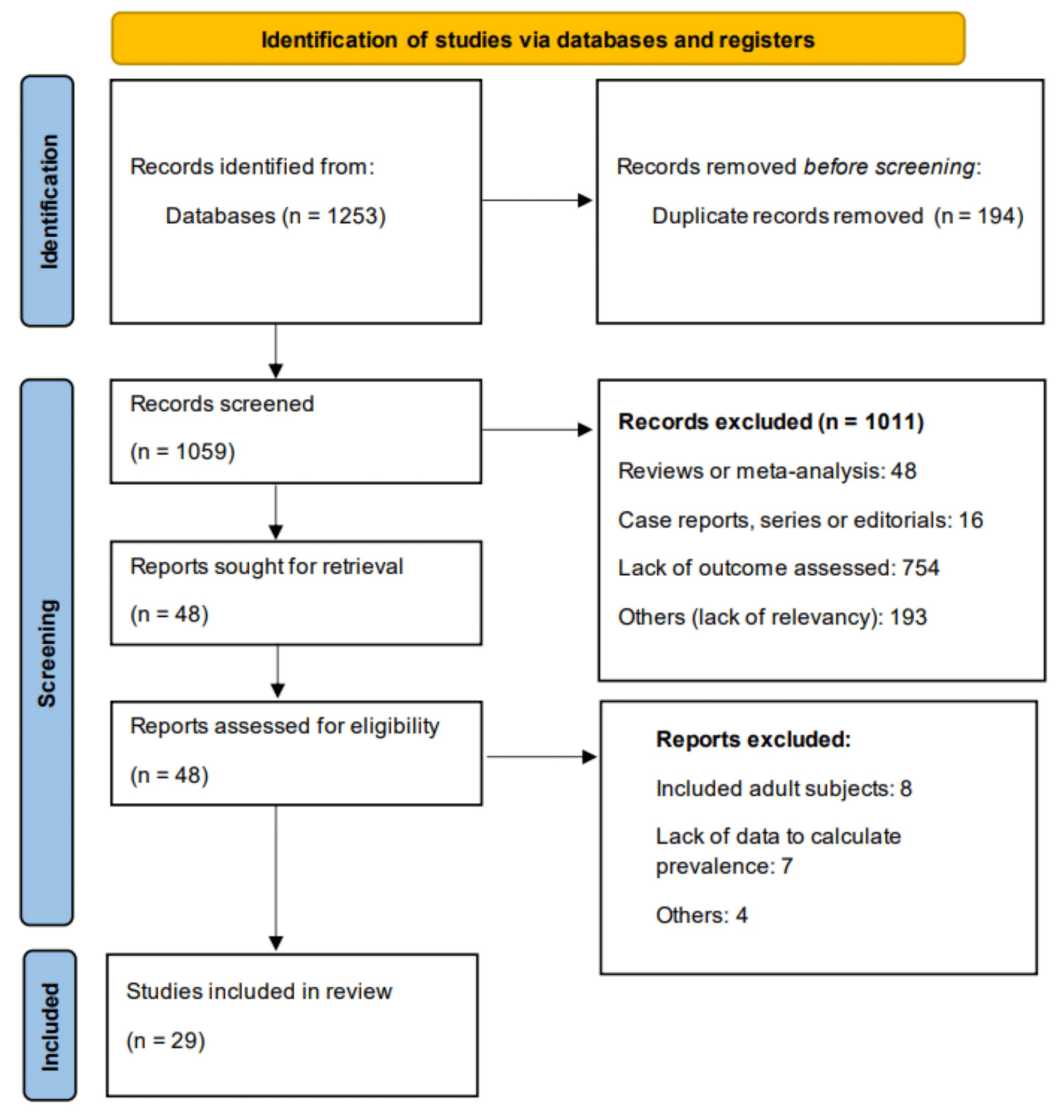
PRISMA flowchart (Study selection process) PRISMA: Preferred Reporting Items for Systematic Reviews and Meta-Analyses

We retrieved 1253 records from online databases. After removing 194 duplicates, 1059 were screened using their titles and abstracts. Through this process, we obtained 48 studies. Full-text of these studies was obtained and detailed assessment was done based on pre-defined inclusion and exclusion criteria. Ultimately, 29 studies were included in this meta-analysis. Figure 1 shows the PRISMA flowchart of the selection process. 

The analysis included 29 studies, comprising 14 cross-sectional and 14 case-control studies, with a total of 2516 participants. The sample sizes in these studies varied significantly, ranging from 29 to 395 individuals. Among the included participants, a substantial proportion were children and adolescents diagnosed with type 1 diabetes (T1D) and vitamin D deficiency. Most studies defined vitamin D deficiency using thresholds between <10 ng/mL and <30 ng/mL, with common measurement techniques including high-performance liquid chromatography (HPLC), radioimmunoassay (RIA), electrochemiluminescence, enzyme-linked immunosorbent assay (ELISA), and chemiluminescent assays. The highest number of studies came from Saudi Arabia (five), followed by Korea, Iraq, Iran, Italy, and Spain, each contributing two studies. Other countries represented in the dataset included Libya, the United States, Ukraine, Kuwait, the United Kingdom, Turkey, China, Indonesia, Tunisia, and Poland, each with one study. The characteristics of these studies, including vitamin D measurement methods, deficiency cut-off values, and demographic data, are detailed in Table 1. Table 2 presents the quality assessment of included studies. 

**Table 1:** Characteristics of the selected studies HPLC: High-Performance Liquid Chromatography; RIA: Radioimmunoassay; ELISA: Enzyme-Linked Immunosorbent Assay; T1D: Type 1 Diabetes; VDR: Vitamin D Receptors; LC-MS/MS: Liquid Chromatography-Tandem Mass Spectrometry; EIA: Enzyme Immunoassay; ELFA: Enzyme-Linked Fluorescent Assay; ECLIA: Electrochemiluminescence Immunoassay; NR: Not Reported; HB1AC: Hemoglobin A1C; n: Number of subjects

Author	Region	Study design	Sample	Subjects with Vitamin D deficiency (n)	Continent	Vitamin D Measure	Deficiency cut-off	Mean age (Years)	Males (n)	HbA1C
Alasbily et al., 2024 [14]	Libya	Cross-sectional	63	17	Asia	NR	<10 ng/ml	11	NR	10.1
Alkharashi et al., 2019 [15]	Saudi Arabia	Cross-sectional	100	70	Asia	Biochemical	<15 ng/mL	NR	50	8.6
Almansour et al., 2025 [16]	Saudi Arabia	Cross-sectional	218	120	Asia	ELISA	<20 ng/ml	12	117	9.2
Al Sawah et al., 2016 [17]	United States	Cross-sectional	197	80	America	(LC-MS/MS)	<20 ng/ml	NR	112	8.6
Al Shaikh., 2016 [18]	Saudi Arabia	Cross-sectional	301	103	Asia	Chemiluminescent assay	<15 ng/mL	13.9	140	9.67
Al-Zubeidi 2016 [19]	Saudi Arabia	Cross-sectional	185	33	Asia	Chemiluminescent assay	<20 ng/ml	9.8	81	NR
Bae et al., 2018 [20]	Korea	Case-control	85	41	Asia	RIA	<20 ng/ml	14.5	37	NR
Biliaieva et al., 2022 [21]	Ukraine	Case-control	94	64	Asia	Electrochemiluminescence	<20 ng/ml	NR	NR	NR
Carakushansky et al., 2020 [22]	United States	Cross-sectional	395	17	America	(LC-MS/MS)	<15 ng/mL	12.91	202	NR
Elsayed et al., 2016 [23]	Kuwait	Cross-sectional	42	14	Asia	ELISA	<12 ng/ml	NR	NR	NR
Federico et al., 2018 [24]	Italy	Case-control	82	41	Europe	HPLC	<20 ng/ml	9.4	44	10.35
Giri et al., 2017 [25]	United Kingdom	Cross-sectional	271	40	Europe	Tandem Mass Spectrometry	<12 ng/ml	7.7	NR	8.5
Hafez et al., 2017 [26]	Egypt	Cohort	49	35	Africa	NR	<12 ng/ml	10.2	27	9
Haleem, 2019 [27]	Iraq	Case-control	50	45	Asia	HPLC	<10 ng/ml	NR	27	NR
Hassan et al., 2016 [28]	Egypt	Cross-sectional	60	55	Africa	Chemiluminescent assay	<20 ng/ml	9.1	NR	NR
Kim et al., 2017 [29]	Korea	Case-control	42	24	Asia	I-labeled radioimmunoassay	<20 ng/ml	NR	12	NR
Kor et al., 2018 [30]	Turkey	Cross-sectional	106	7	Asia	Chemiluminescent assay	<12 ng/ml	13.35	44	12.82
Liu et al., 2018 [31]	China	Case-control	296	39	Asia	EIA	<12 ng/ml	8.66	147	NR
Mansi et al., 2021 [32]	Iraq	Case-control	104	83	Asia	NR	<25 ng/ml	3.72	36	
Polat et al, 2022 [33]	Turkey	Case-control	29	16	Asia	NR	<30 ng/ml	NR	NR	NR
Rasoul et al 2016 [9]	Kuwait	Case-control	216	182	Asia	EIA	<20 ng/ml	NR	104	NR
Rochmah et al., 2022 [34]	Indonesia	Case-control	31	4	Asia	ELFA	<20 ng/ml	11.22	18	NR
Saki et al., 2016 [35]	Iran	Cross-sectional	85	17	Asia	HPLC	<20 ng/ml	12.4	39	10.2
Savastio et al 2016 [36]	Italy	Longitudnal	64	41	Europe	Chemiluminescent assay	<10 ng/ml	7.7	NR	11.4
Segovia-Ortí et al.,2020 [37]	Spain	Cross-sectional	67	13	Europe	Chemiluminescent assay	<20 ng/ml	NR	31	11.68
Sonia et al., 2016 [10]	Tunisia	Case-control	29	15	Africa	RIA	<20 ng/ml	8.95	14	10.3
Wierzbicka et al 2016 [8]	Poland	Case-control	60	49	Europe	ECLIA	<20 ng/ml	15.3	28	7.9
Zambrana-Calvi et al., 2016 [38]	Spain	Cross-sectional	90	12	Europe	HPLC	<15 ng/mL	11.7	46	7.5
Ziaei-Kajbaf et al., 2018 [39]	Iran	Case-control	85	65	Asia	ELISA	<20 ng/ml	8.73	40	NR

**Table 2:** Quality assessment of the included studies

Study ID	Study design	Overall	Rating
Alasbily et al., 2024 [14]	Cross-sectional	10	Good
Alkharashi et al., 2019 [15]	Cross-sectional	10	Good
Almansour et al., 2025 [16]	Cross-sectional	10	Good
Al Sawah et al., 2016 [17]	Cross-sectional	9	Good
Al Shaikh., 2016 [18]	Cross-sectional	10	Good
Al-Zubeidi 2016 [19]	Cross-sectional	10	Good
Bae et al., 2018 [20]	Case-control	8	Good
Biliaieva et al., 2022 [21]	Case-control	6	Fair
Carakushansky et al., 2020 [22]	Cross-sectional	11	Good
Elsayed et al., 2016 [23]	Cross-sectional	8	Good
Federico et al., 2018 [24]	Case-control	7	Good
Giri et al., 2017 [25]	Cross-sectional	8	Good
Hafez et al., 2017 [26]	Cohort	8	Good
Haleem, 2019 [27]	Case-control	7	Good
Hassan et al., 2016 [28]	Cross-sectional	8	Good
Kim et al., 2017 [29]	Case-control	8	Good
Kor et al., 2018 [30]	Cross-sectional	5	Fair
Liu et al., 2018 [31]	Case-control	7	Good
Mansi et al., 2021 [32]	Case-control	8	Good
Polat et al, 2022 [33]	Case-control	7	Good
Rasoul et al 2016 [9]	Case-control	9	Good
Rochmah et al., 2022 [34]	Case-control	8	Good
Saki et al., 2016 [35]	Cross-sectional	10	Good
Savastio et al 2016 [36]	Longitudnal	10	Good
Segovia-Ortí et al.,2020 [37]	Cross-sectional	9	Good
Sonia et al., 2016 [10]	Case-control	8	Good
Wierzbicka et al 2016 [8]	Case-control	8	Good
Zambrana-Calvi et al., 2016 [38]	Cross-sectional	10	Good
Ziaei-Kajbaf et al., 2018 [39]	Case-control	8	Good

Meta-Analysis on the Proportion of Children or Adolescents With T1DM 

**Figure 2:**
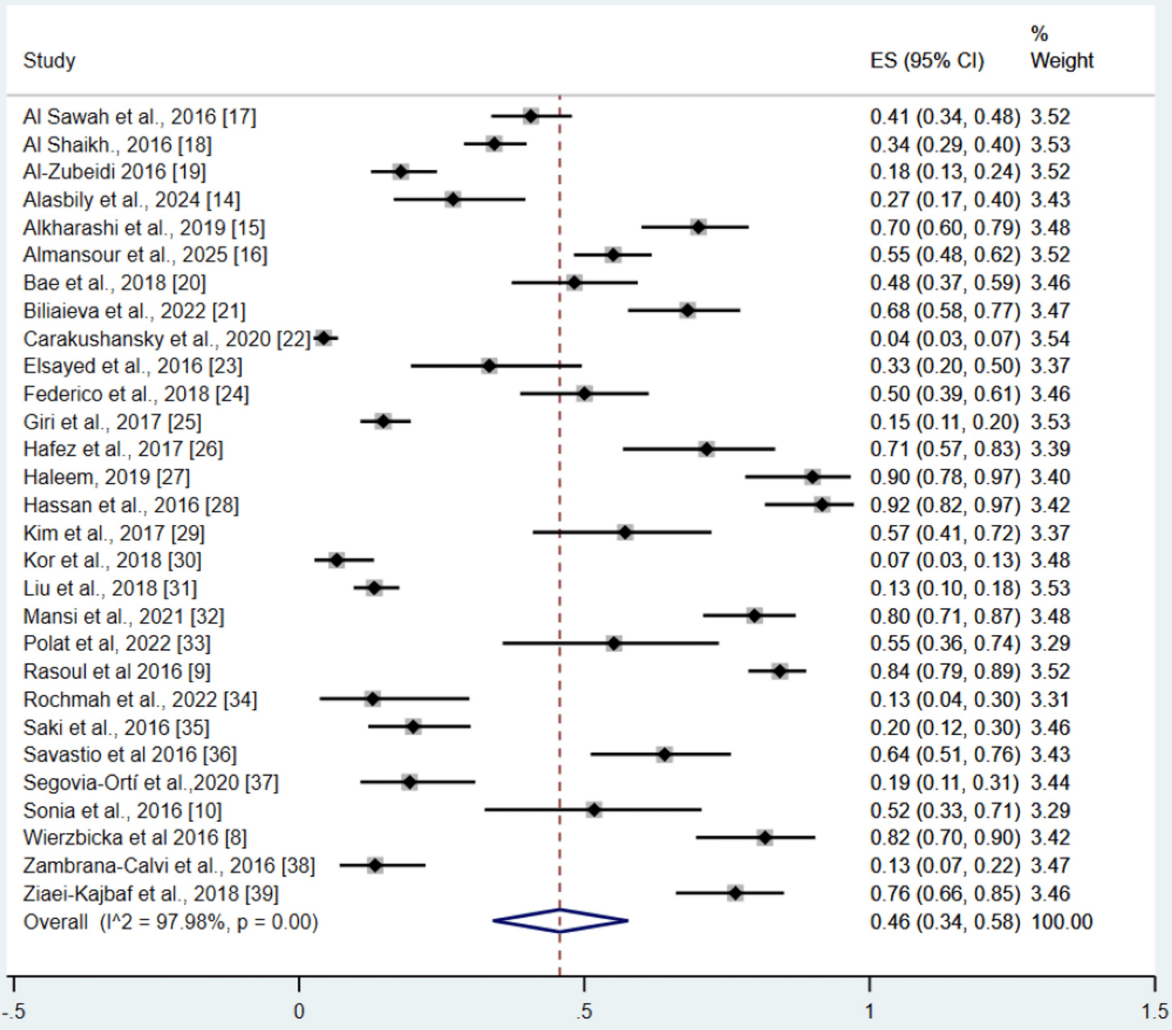
Forest plots for the total proportion of vitamin D deficiency in children/adolescents with type 1 diabetes References [8-10, 14-39]

Pooled analysis of 29 studies showed that the proportion of deficiency of vitamin D in children or adolescents with T1DM was 46% (95% CI: 34 to 58%). Analysis showed high heterogeneity between studies with I-Square value of 97.98% and p-value<0.01 as shown in Figure 2. Publication bias was assessed using the Egger’s test. Publication bias was observed as Egger’s test p-value was less than 0.001. We performed subgroup analysis on the basis of publication year, study design, geographical region, and vitamin D deficiency classification; the results are presented in Table 3. The subgroup analysis revealed notable variations in the proportion of vitamin D deficiency across different study characteristics. 

**Table 3:** Subgroup Analysis CI: Confidence interval; NA: Not applicable

Subgroup	Categories	Number of Studies	Proportion (95% CI)	I-Square
Publication year	Before 2020	21	48 (34 to 62)	98.91%
	2020 onwards	9	41 (18 to 66)	98.64%
Geographical Region	Asia	18	47 (33 to 62)	98.61%
	Europe	6	40 (18 to 62)	97.77%
	Africa	3	74 (48 to 93)	NA
	America	2	13 (10 to 16)	NA
Study Design	Case-control	13	59 (40 to 78)	98.51%
	Cross-sectional	14	30 (18 to 44)	97.71%
	Longitudnal or Cohort	2	64 (51 to 76)	NA
Vitamin Deficiency Classification	<10 ng/ml	3	60 (23 to 98)	NA
	<12 ng/ml	5	14 (8 to 20)	80.51%
	<15 ng/ml	4	30 (5 to 55)	97.61%
	<20 ng/ml	15	49 (34 to 63)	97.61%
	<25 ng/ml	1	80 (71 to 87)	NA
	<30 ng/ml	1	55 (36 to 74)	NA

Studies conducted before 2020 reported slightly higher deficiency rates compared to those from 2020 onwards (48% (95% CI: 34 to 62%) vs. 41% (95% CI: 18 to 66%)), both with high heterogeneity (I² = 98.91% and 98.64%, respectively). Regionally, Africa had the highest deficiency rate (74% (95% CI: 48 to 93%)), followed by Asia (47% (95% CI: 33 to 62%)), Europe (40% (95% CI: 18 to 62%)), and America with the lowest (13% (95% CI: 10 to 16%)). 

Regarding study design, longitudinal or cohort studies showed the highest deficiency rate (64% (95% CI: 51 to 76%)), followed by case-control studies (59% (95% CI: 40 to 78%)), and cross-sectional studies (30% (95% CI: 18 to 44%)). Deficiency classification thresholds significantly influenced estimates, with the <25 ng/mL cutoff yielding the highest proportion (80% (95% CI: 71 to 87%)), while the <12 ng/mL threshold yielded the lowest (14% (95% CI: 8 to 20%)). Overall, significant heterogeneity was observed across most subgroups, highlighting variability in study populations and methodological differences.

Discussion 

This meta-analysis assessed the prevalence of vitamin D deficiency in children or adolescents with T1DM. The pooled estimate of 29 studies showed that the proportion of vitamin D deficiency in children or adolescents with T1DM was 44% (95% CI: 33 to 55%). These findings can help enhance interventions targeting children or adolescents to decrease the prevalence of vitamin D deficiency in T1DM. Furthermore, these results serve as a warning that vitamin D deficiency requires more attention in clinical settings.

The notably high rate of vitamin D deficiency observed in children and adolescents with type 1 diabetes may be attributed to the nature of vitamin D as a fat-soluble compound. Its primary absorption occurs in the small intestine, followed by metabolic conversion in the skin, liver, and kidneys to its active form, 1,25-dihydroxyvitamin D. Efficient uptake of fat-soluble vitamins relies on complex physiological mechanisms, which not only require a healthy intestinal lining but also depend on external digestive components such as pancreatic lipase and bile acids produced by the liver [40].

The pooled analysis reported high heterogeneity among the study results. We performed subgroup analysis based on different variables, including publication year, study design, geographical location, and vitamin D deficiency classification. The results of subgroup analysis by publication year did not show any difference in the proportion of vitamin D deficiency. Moreover, when evaluating the prevalence of vitamin D deficiency across various study designs, this analysis revealed a higher deficiency rate in case-control studies compared to other types. This variation highlights how the choice of study design can significantly influence the estimated proportion of individuals with vitamin D deficiency.

A recent meta-analysis investigated the association between serum vitamin D levels and type 1 diabetes in children. It reported significantly lower vitamin D levels in patients compared to healthy controls [41]. Another analysis estimated that approximately 45% (95% CI: 37-54%) of children and adolescents with T1D were vitamin D deficient [4]. Consistent with previous findings, our study also demonstrates a high prevalence of vitamin D deficiency among individuals with T1DM. However, several important questions remain unanswered: (a) What should be considered the optimal serum 25(OH)D level for patients with T1DM? and (b) Can vitamin D supplementation or its analogs play a preventive or therapeutic role in managing T1DM, and is there an immediate need for such supplementation? Addressing these uncertainties will require well-designed, large-scale, long-term clinical trials.

This study has several limitations that should be considered. First, all included studies were conducted in clinical or hospital settings, which may not accurately reflect the true prevalence of vitamin D deficiency in the broader population of children and adolescents with type 1 diabetes. Second, the analysis incorporated a range of study designs, including cross-sectional, case-control, cohort, and longitudinal studies, each of which carries inherent methodological limitations and potential biases. Third, there is currently no universally accepted definition or threshold for vitamin D deficiency, leading to variability in how deficiency is classified and reported across studies. Lastly, although Egger's test was used to assess publication bias, its presence cannot be entirely ruled out.

Emerging evidence suggests that adequate intake of vitamin D may play a beneficial role in the management and possibly prevention of T1DM [42-43]. Vitamin D has immunomodulatory effects that may help regulate autoimmune responses implicated in the destruction of pancreatic β-cells, a hallmark of T1DM [44]. By promoting immune tolerance and reducing inflammation, vitamin D may slow the progression of β-cell damage in individuals at risk or in the early stages of the disease. Moreover, some studies have shown that vitamin D supplementation is associated with improved glycemic control, possibly by enhancing insulin sensitivity and preserving residual insulin secretion [45]. While these findings are promising, further randomized controlled trials are needed to establish optimal dosing strategies, timing of intervention, and long-term outcomes in children and adolescents with T1DM.

## Conclusions

Our meta-analysis reveals that vitamin D deficiency is highly prevalent among children and adolescents with type 1 diabetes mellitus, affecting approximately 46% of this population. This substantial burden highlights the need for increased clinical awareness and potentially routine screening in pediatric T1DM patients. However, several questions remain unanswered, including optimal serum 25(OH)D levels for T1DM patients and the therapeutic potential of vitamin D supplementation. Further research, particularly well-designed randomized controlled trials, is needed to establish evidence-based guidelines for vitamin D management in this vulnerable population and to determine whether supplementation could help prevent complications or improve outcomes in pediatric T1DM.
